# Prevalence Rate of Adolescent Idiopathic Scoliosis: Results of School-based Screening in Surabaya, Indonesia

**DOI:** 10.5704/MOJ.1711.011

**Published:** 2017-11

**Authors:** IS Komang-Agung, SB Dwi-Purnomo, A Susilowati

**Affiliations:** Department of Orthopaedics, Airlangga University, Surabaya, Indonesia; * Department of Public Health & Community Medicine, Airlangga University, Surabaya, Indonesia

**Keywords:** school-based scoliosis screening, children, prevalence rate

## Abstract

**Introduction:** Scoliosis is a lateral spinal deformity of 10 degrees or more, resulting in a C-shaped or S-shaped curve of the spine. Information about adolescent idiopathic scoliosis (AIS) prevalence rate is important not only for paediatric health care planning strategy but also for parent’s awareness. This study aims to find the suitable inclination cut-off angle and the prevalence rate of AIS in Surabaya, Indonesia.

**Materials and Methods:** This is a descriptive cross-sectional study conducted in 2010. We performed stratified random sampling of 784 Elementary and Junior High School students in Surabaya between 9-16 years of age. Scoliosis screening was performed by the Adam’s forward bending test (FBT). The students with positive FBT were measured for the inclination angle with scoliometer, and then subjected to radiologic examination. Prevalence rate, gender ratio, and the cut-off point value of inclination angle were determined by a descriptive statistics analysis.

**Results:** Adam’s forward bending test was positive in 50 students (6,37%). Among them, 23 students (2,93%) four males and 19 females had Cobb angle of ≥10°. The 5° cutoff point value of inclination angle had a 95.6% sensitivity, an 18.5% specificity, a 50% positive predictive value (PPV), and a 83.33% negative predictive value (NPV); while the 7° cut-off point had a 78.26% sensitivity, a 88.88% specificity, a 85.7% PPV, and a 82.7% NPV.

**Conclusion:** The prevalence rate of AIS in Surabaya is 2.93% and the 7° cut-off point of inclination angle is suitable for school-based screening.

## Introduction

Idiopathic scoliosis is commonly found in children of age 10 years or older, most of the time not realized or noticed until too late^2,3,4^. The deformity could break one’s self-esteem especially in the adolescent age. There is no clear etiology of idiopathic scoliosis. Among the many theories, there are genetic factors, connective tissue and skeletal muscle abnormality, and biomechanical factors that may play roles in its development. The clinical manifestation of AIS could range from asymptomatic or minor complaints to major cardiopulmonary and neurological symptoms^3^.

Early detection of AIS and proper management are the keys to satisfactory results. Screening on school-age children is an effective way to detect scoliosis earlier. Thus, interventions either non-surgical (bracing or restoring the possible life style which interfere the abnormal biomechanical factors) or surgical, can be conducted earlier to prevent scoliosis progression^1,2,5,6^.

In Indonesia, scoliosis management is often delayed due to a lack of awareness among patients and parents. There are many ways to conduct the scoliosis screening such as: Adam’s forward bending test, plumb line test, or measurement using the scoliometer. The scoliometer is used to measure the magnitude of vertebral rotation to its axis^6,7,8^. Some countries, like the USA and Hong Kong, report that the school-based screening program is costly and inefficient; that the effort to detect one case when surgery was indicated would require 450 student to be screened and around 20% (90 students) to undergo radiological examination^9^.

The prevalence rate varies worldwide according to country and ethnicity. It also depends on the cut-off scoliosis criteria in the screening protocol, which is 0.5 % (20° Cobb angle) -7 % (10° Cobb angle) ^8^. Every country should determine its own prevalence rate, and especially so in a multiracial country like Indonesia. To achieve this objective, we were required to devise a good screening protocol. The aims of this study were to determine the AIS prevalence rate in Surabaya, Indonesia, the gender ratio and the cut-off point value of inclination angle which suited our sociodemographic for an effective screening protocol.

## Materials and Methods

This is a descriptive, cross sectional and population-based study, in collaboration with the regional Ministry of Health and Ministry of Culture and Education, approved by the local orthopaedic research and ethics committee. According to the 2010 National Population Census, the population aged 5 to 19 years old in Surabaya City was 649,816^10^. The study population was made up of students from elementary and junior high schools in Surabaya in July 2010. Consent was obtained from the students and their parents prior to the screening.

The sample was acquired using stratified random sampling according to the proportion of the total elementary and junior high school students throughout Surabaya City, based on the data provided by the local Education Authority of Surabaya in 2010. Surabaya is divided into five regions of North, West, East, South, and Central. From each region, two districts were picked randomly, then the sampling of elementary and junior high schools were conducted randomly according to the proportion of schools in the district. One out of ten elementary schools, and one out of five junior high schools were selected. The authorities in the chosen elementary and junior high schools were asked to allow their students, aged 9 years or older, to participate in the scoliosis screening.

The sample size for this study was determined using descriptive study sample size calculation formulated as follows:

**Figure:**
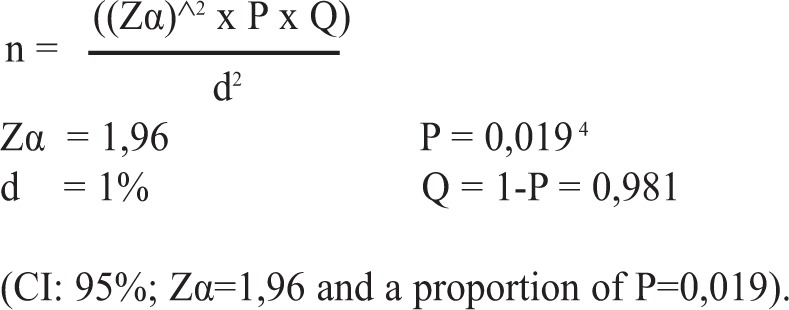


The proportion value was determined according to the results of the scoliosis prevalence rate study conducted by Shands and Eisberg in Delaware^11^. The precision rate was d=1%, the minimum sample for the screening was 716 students.

The Adam’s forward bending test (FBT) was applied for the scoliosis screening. The test was considered positive when back asymmetry (presence of hump) was detected. Further measurement of inclination degree using scoliometer was conducted when FBT was positive. Students were then radiologically examined to measure the Cobb angle. The student was diagnosed with scoliosis when the Cobb angle was 10° or more.

All radiological examinations were conducted in Surabaya Orthopedic & Traumatology Hospital due to availability and accessibility. The data results were statistically analyzed using SPSS version 17.0 for Windows to determine the prevalence rate, the gender ratio, and the cut-off point of inclination angle. The validity (sensitivity and specificity) of Adam’s forward bending test and the minimum inclination degree by scoliometer was calculated upon the confirmed diagnosis from radiologic examination as the gold standard (Cobb angle of ≥ 10°).

## Results

The total number of participants who went through scoliosis screening was 784 students from elementary and junior high schools. The gender distribution were 315 male students (40.2%) and 469 female students (59.8%) (Table I). The ethnic denomination of the sample students was 770 Melayu, ten Chinese, one Melanesian (Maluku origin), and three Weddoid (Papua origin). The age distribution of the scoliosis screening participants was between 9 to 16 years old, with an average of 12.61 years and a median of ten years (24.1%). Fifty Melayu students were found to have asymmetrical back (hump detected, resulting in a positive Adam’s forward bending test (6.4%). The positive test was found in 14 males (1.8%) and 36 females (4.6%). The age distribution of positive FBT was mostly (11 cases each) consisting of students aged 10 years old, 14 years old, and 15 years old. The inclination degree (5°-7°) measured with scoliometer was found in 23 students, whereas 21 students were measured with a ≥ 7° of inclination. Despite the 44 students measuring ≥ 5°; all 50 FBT positive students were further subjected to radiological examination and two candidates were found to have Cobb angle 10° and 12° (false negative).

**Table I:** Demographic profile of students

Age	Gender		Screening test results (FBT)				Cobb angle >10°	
	Male	Female	Negative		Positive			
			n	%	n	%	n	%
9	5	7	11	1.4	1	0.13	0	0
10	90	100	179	22.83	11	1.4	3	0.38
11	41	54	91	11.61	4	0.51	3	0.38
12	29	25	51	6.5	3	0.38	0	0
13	28	58	83	10.58	3	0.38	2	0.26
14	77	111	177	22.58	11	1.4	5	0.06
15	40	75	104	13.27	11	1.4	8	0.10
16	5	39	38	4.85	6	0.77	2	0.02
Total	315	469	734	93.62	50	6.37	23	2.93

Radiological examination for Cobb angle with ≥10° were found in 23 students (prevalence rate 2.93%); four males, and 19 females (Table II). The ratio of male to female scoliosis cases was 1: 4.7. Fifteen students (1.91%) had Cobb angle of 10°-20°; five students (0.64%) had Cobb angle of 20°- 40° and three students (0.38%) had Cobb angle of >40° (severe scoliosis); one student with > 50° and two students with >110°. All severe scoliosis cases were females and 14 years of age.

**Table II:** Gender proportion of students with scoliosis

		Scoliosis (+) n	Prevalence (%)
Gender	Male	4	0,51
	Female	19	2,42
	Total	23	2,93
Cobb Angle	10° - 19°	15	1,91
	20°- 40°	5	0,64
	>40°	3	0,38
	Total	23	2,93

The sensitivity test for the cut-off inclination angle of 5° was 91.3% sensitivity, 18.5% specificity, 71.4% negative predictive value and 48.8% positive predictive value. The 7° cut-off point had 78.3% sensitivity, 88.9% specificity, 82.7% NPV and 85.7% PPV (Table III).

**Table III:** Validity test for different inclination cut-off angle

Inclination	Cobb Angle ≥10° (Scoliosis +)	Cobb Angle ≥10° (Scoliosis -)	Total	
≥ 5°	21	22	43	
< 5°	2	5	7	
Total	23	27	50	
Sensitivity				91.3%
Specificity				18.5%
PPV				48.8%
NPV				71.4%
≥ 7°	18	3	21	
< 7°	5	24	29	
Total	23	27	50	
Sensitivity				78.3%
Specificity				88.9%
PPV				85.7%
NPV				82.7%

## Discussion

In this study three out of 784 students were found to have severe scoliosis, all female and in the age of 14 years. We could presume that if only this screening had been held three years earlier, the disease might not have progressed to a more severe condition which could only be treated by surgery. Three cases out of 784 is quite significant and worthy of the screening effort compared to the Minnesota study (1 out of 450 screened children)^12^. American Academy of Orthopaedic Surgeons has released a position statement that screening for spinal deformity should be part of the medical home preventive services visit. The screening is considered valuable in these domains: technical efficacy, clinical program, and treatment effectiveness. It should be aimed at identifying suspected cases of scoliosis to be considered as positive cases and which would require to be referred for further evaluation^14^. A study in Guangdong, China, suggested that it is important to screen schoolchildren in age groups 12 to 13, 13 to 14, 14 to 15, and 15 to 16 years for a significantly high prevalence^15^. They believed that earlier identification will benefits the children and information should be extended to patients and parents, health care providers and policy makers. The value of school-based scoliosis screening programs is well established despite some controversial issues, such as the relatively low prevalence of significant curves in the general population, the lack of a definitive diagnostic screening test, as well as the cost-effectiveness of the screening program. However, the screening program are proven to be beneficial in the study by Mukesh *et al* to identify children at risk for several health problems related to scoliosis^16^. A study investigating the clinical effectiveness of school screening program in Malaysia showed a significant positive predictive value reflecting a significant amount of the percentage of students diagnosed with scoliosis amongst those positively screened using scoliometer. This meant that it was adequate to suggest that the screening program did play a role in early detection of scoliosis^17^. A cohort study conducted in Hong Kong also supported the program and recommended its own cohort study to continue^9^.

**Fig. 1:**
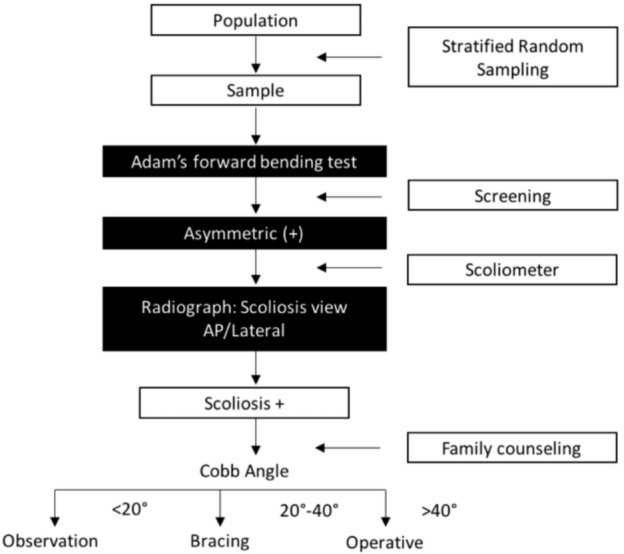
Screening protocol of AIS in Surabaya, Indonesia.

**Fig. 2:**
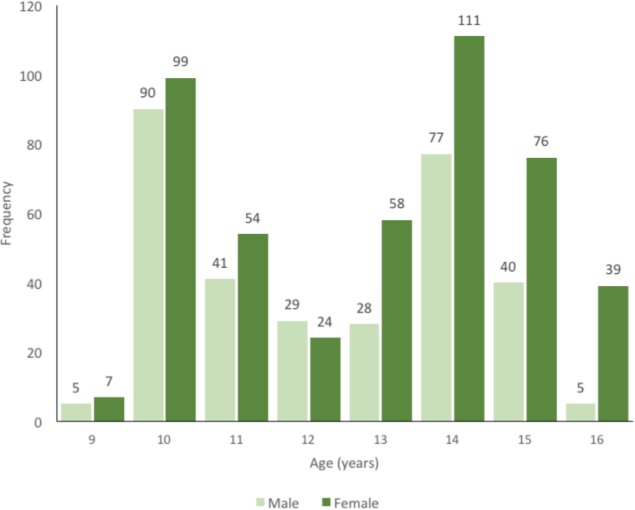
Scoliosis screening age and sex distribution.

The ideal test tool is one that has a high sensitivity and specificity, but the predictive factor is more susceptible for a screening test since a false negative result will give worse impact for the population. The person who had a false negative result would lose the opportunity to be treated. False positive would invite stressful further examination. Scoliometer was chosen as it is currently the best tool for scoliosis screening^14^. As stated by the American Academy of Orthopaedic Surgeons (AAOS), the forward bend Adam’s test with the use of a scoliometer should be used for the screening^13^, the same method as was used in this study.

The meta-analysis study of the effectiveness of the screening program suggests that programs that used the FBT as the only screening tool had a higher referral rate and a lower precision in detecting scoliotic curves^18^; hence, the combination of FBT and scoliometer in the present study. We therefore suggest making the combination of FBT and scoliometer as a screening tool mandatory. The students suspected to have scoliosis based on the screening would be referred for further diagnostic evaluation to be ruled out or confirmed as having clinically significant scoliosis^14^.

In this study, the 5° cut-off point was sensitive though other indicator showed bad predictive value; confirmed by the bad correlation with the 10° Cobb angle (Table III). Only three out of 22 positives > 5° that were confirmed to have Cobb angle > 10°. While 18 out of 21 positives > 7° were confirmed for the >10° Cobb angle. Many centers used 5° or 7° cut-off point considering many aspects, including using both cut-off; 7° for normal BMI and 5° for overweight children^9,17^. The missed opportunity to be treated might further lead to disbelief in the screening test. The unnecessary cost for further test and radiography exposure also need to be considered. We decided to use 7° cut-off point for the inclination value based on these findings.

The present study revealed a scoliosis prevalence of 2.93% in school-age children between 9-16 years old in Surabaya, which is high compared to other Asian country study (1.09% in Nepal; 2.22% in Singapore), and also when compared with the Minnesota study in 1977 of 1.1%^12^. Adolescence Idiopathic Scoliosis (AIS) is common with an overall prevalence of 0.47-5.25^19^. The AIS ratio of male to female in this study was 1: 4.7. In the study in Chiba, Japan, the male to female ratio was 1: 3.7^7^. Based on these findings and what the SRS International Task Force on Scoliosis Screening recommends, male students should be screened once at age 13 or 14, whereas females should be screened twice at age 10 or 12^14^.

The authors noted some limitations of this study. The inter-observer bias was possible when conducting the FBT and measuring the inclination degree with scoliometer. The sample size in this study was not large enough and did not represent the wide variety of races in Indonesia; although all results were comparable with studies in other countries. The authors recommend regular screening of AIS not only in Surabaya but other parts of Indonesia to obtain national figures. The data collected from a large population based screening could be used to perform longitudinal study in which bias could be minimized.

## Conclusion

The prevalence rate of adolescent idiopathic scoliosis in school-age children between 9-16 years old in Surabaya, Indonesia, is 2.93%, with male to female ratio of 1: 4,7. The study also found that the inclination degree of 7° or greater is more acceptable compared to inclination degree of 5°.
